# Paper-Based Analytical Devices Coupled with Fluorescence Detection and Smartphone Imaging: Advances and Applications

**DOI:** 10.3390/s26031012

**Published:** 2026-02-04

**Authors:** Constantinos K. Zacharis

**Affiliations:** Laboratory of Pharmaceutical Analysis, Department of Pharmacy, Aristotle University of Thessaloniki, 54124 Thessaloniki, Greece; czacharis@pharm.auth.gr; Tel.: +30-2310-997663

**Keywords:** paper-based analytical device, fluorescence, smartphone detection, determination

## Abstract

Paper-based analytical devices have emerged as a versatile and cost-effective platform for on-site chemical and biological analysis. The integration of fluorescence detection with smartphone imaging has significantly enhanced the analytical performance and portability of these systems, enabling sensitive, rapid, and user-friendly detection of diverse analytes. This review highlights recent advancements in paper-based fluorescence sensing technologies, focusing on their design principles, materials, and detection strategies. Emphasis is placed on the use of nanomaterials, quantum dots, and carbon-based fluorophores that improve sensitivity and selectivity in food, bioanalytical, and environmental applications. The role of smartphones as optical detectors and data processing tools is explored, underscoring innovations in image analysis, calibration algorithms, and app-based quantification methods.

## 1. Introduction

Paper-based analytical devices (PADs) have emerged over the past two decades as powerful tools for low-cost, rapid, and decentralized chemical and biological analysis. Owing to their inherent advantages such as affordability, portability, ease of fabrication, and minimal reagent consumption, PADs have attracted significant attention for applications in clinical diagnostics, environmental monitoring, food safety, and point-of-care (POC) testing [1,2,3,4,5]. The ability of paper substrates to transport fluids via capillary action without external pumps further enhances their suitability for resource-limited and field-deployable settings [6].

Among the various detection strategies integrated with PADs, fluorescence-based detection has gained prominence due to its high sensitivity, wide dynamic range, and compatibility with diverse chemical probes and biosensing schemes. Fluorescent labels, nanomaterials, and molecular sensors enable the selective and quantitative detection of a broad range of analytes, including ions, small molecules, nucleic acids, proteins, and pathogens [7]. When coupled with paper platforms, fluorescence detection can significantly improve analytical performance while maintaining the simplicity of device operation. However, conventional fluorescence instrumentation is often bulky, expensive, and impractical for on-site analysis, limiting the full potential of PAD-based fluorescence assays.

Recent advances in smartphone technology have provided a transformative solution to the above limitations [8]. Modern smartphones are equipped with high-resolution cameras, powerful processors, and advanced image analysis capabilities, making them attractive alternatives to traditional optical detectors. When integrated with PADs and appropriate excitation sources or optical accessories, smartphones can capture fluorescence signals with remarkable accuracy and reproducibility. This synergy enables quantitative analysis, data storage, wireless transmission, and real-time decision-making, thereby expanding the accessibility and functionality of paper-based fluorescence assays.

**Figure 1:**
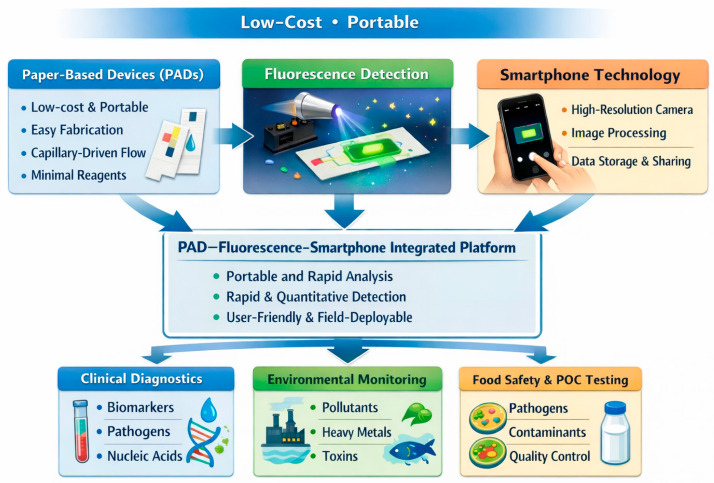
A schematic representation of the key aspects of the present review.

Few review articles have been published on the applications of paper-based devices using a smartphone for detection [9,10]. However, the present review comprehensively examines the advances and analytical applications of paper-based devices coupled with fluorescence detection and smartphone imaging. Key developments in paper substrates, fluorescence probes, device architectures, and smartphone-based imaging and data processing are discussed. Representative applications across biomedical diagnostics, environmental analysis, and food safety are highlighted, along with current challenges related to sensitivity, standardization, and robustness. A schematic representation of the aspects of the present review is shown in Figure 1. Finally, emerging trends and future perspectives are outlined, emphasizing the role of PAD–fluorescence–smartphone integration in advancing next-generation POC and field-deployable analytical technologies.

## 2. Fundamentals of PADs

### 2.1. Structure and Materials of PADs

PADs are generally constructed from cellulose-based substrates, including filter paper, chromatography paper, and nitrocellulose membranes, which are selected for their well-defined porosity, intrinsic hydrophilicity, mechanical flexibility, low cost, and broad chemical compatibility with biological and chemical reagents [11,12]. The interconnected fibrous microstructure of these materials forms a porous network that supports capillary-driven flow, enabling liquids to wick spontaneously without the need for external pumps. Fluid transport is governed by surface tension and pore geometry, allowing for precise manipulation of sample volumes and flow rates within hydrophilic pathways. These pathways are spatially confined by hydrophobic barriers that define channels, reaction zones, and detection areas. Hydrophobic patterning is commonly achieved through techniques such as wax printing, inkjet printing of hydrophobic inks, laser treatment, and photolithography, each providing distinct trade-offs in terms of fabrication complexity, spatial resolution, scalability, and reproducibility [13]. The choice of patterning method significantly influences device performance, particularly with respect to channel fidelity, fluid containment, and suitability for mass production.

In its simplest configuration, a PAD is composed of three fundamental functional zones: (i) a sample introduction zone, where the sample is deposited and absorbed into the paper matrix, (ii) a reaction or detection zone, which contains immobilized chemical or biological reagents, probes, or recognition elements that interact with target analytes to generate a measurable signal, and (iii) a wicking or waste zone, which sustains continuous fluid transport by capillary action and serves to collect excess sample, thereby regulating flow and preventing back-diffusion [10,14].

More advanced PAD architectures, including three-dimensional (3D) PADs and multilayer or stacked devices, significantly expand analytical functionality by enabling vertical and sequential fluid transfer between layers. These configurations facilitate complex operations such as multiplexed assays, multistep reactions, controlled timing of reagent delivery, and on-device reagent storage, ultimately enhancing sensitivity, throughput, and assay integration while maintaining simplicity and portability inherent to paper-based platforms [14,15].

### 2.2. Fabrication Techniques

A variety of fabrication strategies have been developed to produce PADs, each aiming to balance patterning precision, material cost, fabrication throughput, and scalability [10]. Wax printing remains the most widely adopted approach due to its operational simplicity, low equipment requirements, and cost-effectiveness. In this method, solid wax is deposited onto the paper substrate and subsequently thermally melted, allowing the wax to penetrate through the full thickness of the paper and form robust hydrophobic barriers that confine fluid flow [16,17].

Inkjet printing offers enhanced spatial resolution and versatility by enabling the direct deposition of functional inks, including enzymes, nanoparticles, conductive materials, and fluorophores, thereby supporting both fluidic patterning and localized reagent immobilization [18,19]. Screen printing and laser cutting are particularly advantageous for high-throughput and large-area manufacturing, making them well suited for commercial-scale production and integration of PADs into POC testing platforms.

**Figure 2:**
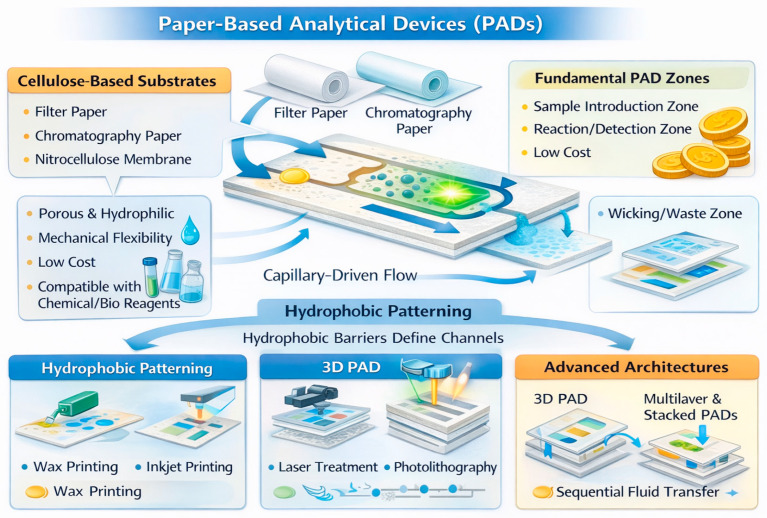
A schematic representation of PADs showing cellulose-based substrates, capillary-driven fluid flow through hydrophobically patterned channels, the main functional zones (sample, reaction/detection, and wicking), and advanced architectures including 3D and multilayer PADs.

More recently, advances in flexible electronics, microcontact printing, and hybrid fabrication techniques have further expanded the versatility of PADs. These approaches enable precise patterning of multiple assay zones, improved control over feature geometry, and seamless integration of electrochemical or optical transducers, thereby supporting the development of multifunctional and instrument-free sensing platforms [20,21,22]. Figure 2 schematically presents various materials and fabrication techniques for PADs.

### 2.3. Optical Compatibility and Background Minimization

Paper’s intrinsic light scattering arising from its fibrous microstructure, along with its autofluorescence from cellulose and residual additives, can significantly interfere with optical detection, particularly under short excitation wavelengths in the ultraviolet to blue region. These background effects often reduce assay sensitivity and limit the accuracy of fluorescence-based measurements on PADs. To mitigate background noise, several strategies have been reported, including the use of long-wavelength fluorophores (e.g., red and near-infrared dyes) to minimize overlap with paper autofluorescence; the adoption of optically transparent or low-autofluorescence paper substrates, such as regenerated cellulose or nanocellulose films; and the implementation of optical filters, ratiometric analysis, and smartphone-based calibration algorithms to enhance signal-to-noise ratios and improve quantitative performance [23,24,25].

In addition, careful optimization of optical paths, including excitation geometry, illumination angle, and emission collection efficiency, is critical for maximizing detection sensitivity and reproducibility in fluorescence-based PADs, particularly when using low-cost imaging systems such as smartphone cameras or portable readers.

Smartphone cameras acquire images through red, green, and blue (RGB) channels, each having different spectral sensitivities that can be exploited for fluorescence detection. The choice of channel is typically guided by the emission spectrum of the fluorophore and the spectral response of the camera sensor, with the channel that best overlaps the emission maximum providing the highest signal-to-background ratio [26]. For example, green-channel analysis is commonly used for fluorophores such as FITC or fluorescein due to their strong emission in the green region, whereas red-channel analysis is preferred for fluorophores emitting at longer wavelengths to minimize background interference from ambient light and autofluorescence [27]. Selecting the optimal channel significantly improves sensitivity by enhancing contrast, reducing cross-talk from other channels, and suppressing noise from non-specific signals. I have added a short summary in the revised manuscript to clarify the rationale behind RGB channel selection and to highlight its direct impact on detection sensitivity and quantitative accuracy in smartphone-based fluorescence measurements.

By tailoring assay design and optical configurations to the characteristics of smartphone cameras, these principles enable sensitive, reproducible, and quantitative signal acquisition using widely available mobile devices. The following section therefore focuses on how these optical considerations are translated into practical smartphone-based detection strategies and system architectures for point-of-care applications.

## 3. Applications

### 3.1. Bioanalysis

PADs have emerged as powerful tools in bioanalysis due to their simplicity, low cost, and suitability for POC applications. By leveraging the intrinsic properties of paper (such as capillary-driven fluid flow, porosity, and ease of chemical modification), PADs enable rapid and sensitive detection of biological analytes without the need for complex instrumentation [28].

An interesting PAD application has been published by I. Ortiz-Gomez et al. for the analysis of carbohydrates [29]. The synthesis of the silicon nanodots was based on a redox reaction between the (3-aminopropyl)triethoxysilane reagent and carbohydrates acting as reducing agents. The authors utilized a heater to increase the temperature of PAD (i.e., 80 °C) in order to fabricate nanodots. The resulting blue-emitting silicon nanodots exhibited a fluorescence emission peak at 475 nm. The method achieved low LOD of 0.80 µM for glucose and 0.51 µM for fructose, along with linear response ranges of 10–200 µM for glucose and 10–100 µM for fructose, respectively. The repeatability of the method was <2.64%. Acceptable recoveries were recorded in the studied biological samples being in the acceptable range of 85–115%. A different approach has been reported in 2021 for the quantitation of glucose in human serum based on N-doped carbon dots and metal oxide hybrid structures [30]. A metal oxide hybrid containing nitrogen-doped CDs was fabricated and utilized as a catalyst to oxidize TMB into its oxidized product in the presence of H_2_O_2_. The fluorescence of the system at 405 nm was quenched upon the addition of H_2_O_2_ due to the combined effects of the IFE and electron transfer among nitrogen-doped carbon dots, and the oxidized TMB, and the glucose. The IFE occurs when the quencher absorbs light that overlaps with the excitation wavelength (primary IFE) or the emission wavelength (secondary IFE) of the carbon dots. As a result, identifying the exact quenching mechanism may be ambiguous in certain situations. Nevertheless, this distinction can be more clearly established by examining changes in fluorescence lifetime and variations in the UV–Vis absorption spectra [31,32]. Using the colorimetric approach, the LODs for H_2_O_2_ and glucose were as low as 84 nM and 0.41 µM, respectively. In comparison, the fluorescence-based method achieved detection limits of 97 nM for H_2_O_2_ and 0.85 µM for glucose. The same reagent was utilized by Wu, Z. et al. to fabricate a dual-mode probe for amoxicillin quantitation [33]. MnO_2_@CQDs catalyzed the oxidation of colorless TMB into its yellow oxidized form. The method was rapid (1 min) and exhibited a linear detection range of 3–45 nM with an LOD at 1 nM.

A red-emissive carbon nanostructure anchored onto a molecularly imprinted metal–organic framework (MOF) was developed as a fluorescent biosensor for the visual detection of dipicolinic acid [34]. This compound is a recognized biomarker of Bacillus anthracis; a pathogen associated with severe infectious diseases and bioterrorism threats [35]. A paper test strip was prepared by coating it with the sensing material. This strip was then combined with UV light for excitation to create a simple fluorescence-based sensing system. In the presence of the analyte, the fluorescence signal decreases, allowing the compound to be detected and measured. The proposed biosensor exhibited a wide linear detection range of 10–125 µM, with LOQ and LOD of 4.32 µM and 1.28 µM, respectively. Owing to its strong emission characteristics and versatile surface functionalization, the sensing platform demonstrated high selectivity toward dipicolinic acid over other biological molecules and structural isomers. As proof of concept, the authors successfully applied their method to the determination of the analyte in real tap water and urine samples.

Nonenzymatic paper-based fluorescent materials (UFP-BP) were fabricated by integrating an on-demand fluorescent sensor for urea detection [36]. The UFP nanoparticles were immobilized onto filter paper, permitting real-time monitoring of the analyte. The synthesized UFP molecules spontaneously self-assembled into fluorescent nanoparticles that exhibit selective recognition of urea. These nanoparticles were then embedded within the interstitial spaces of the filter paper matrix, resulting in the formation of the UFP-BP sensing platform. This material enabled selective and quantitative detection of urea over the range of 10–1000 mM. Both the UFP nanoparticles and the UFP-BP device were successfully applied to urea determination in real rat urine, artificial simulants, and milk samples.

The research group of K. M. Omer developed a portable smartphone-based detection PAD functionalized with green emissive carbon dots (CDs) for selective determination of Fe^3+^ ions in human serum [37]. The CDs were synthesized via a one-step hydrothermal process using two precursors (4-aminophenol, ethylenediamine). The analyte selectively quenched the fluorescence of the CDs. Under optimized conditions and using conventional fluorimetry, the CDs enabled Fe^3+^ detection over a linear range of 0–90 µM (R^2^ = 0.9970), with an LOD of 0.35 µM.

A dual-mode, antidistortion fluorescent sensing strategy on PAD was developed by Qi, J. et al. manipulating the coffee-ring effect to obtain undistorted and quantitative fluorescence images using a smartphone [38]. Through pixel-based RGB analysis and direct measurement of fluorescent strip length, accurate detection of histidine in human urine was achieved with LODs of 0.021 mM and 0.5 mM, respectively. This method enables rapid, sensitive, and convenient smartphone-based fluorescence analysis with improved resistance to image distortion.

Overall, these studies demonstrate the strong potential of PAD-based systems for rapid and sensitive diagnostics; however, challenges related to standardization, long-term stability, and clinical validation must be addressed to enable widespread adoption.

### 3.2. Food Analysis

PADs play an important role in food analysis by providing a low-cost, portable, and easy-to-use platform for rapid testing. They enable on-site detection of contaminants, additives, and nutritional components without the need for complex laboratory equipment or highly trained personnel. Because they require small sample volumes and minimal reagents, these devices are environmentally friendly and suitable for routine screening. Their fast response time and accessibility make them especially valuable for improving food safety monitoring, quality control, and regulatory compliance, particularly in resource-limited settings.

The application of PADs in environmental analysis is summarized in Table 1.

**Figure 3:**
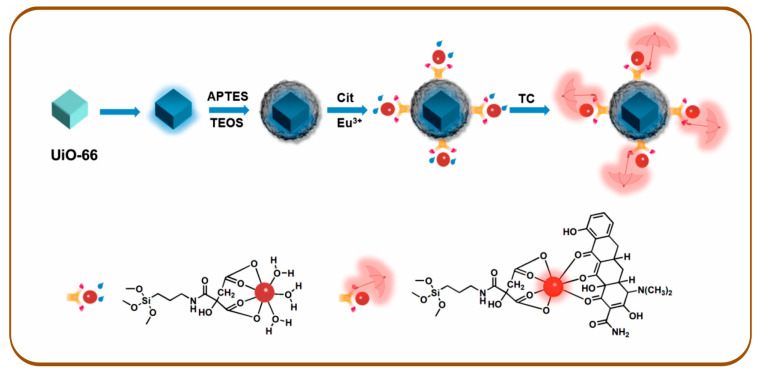
Preparation of the Dye@UiO-66@SiO_2_–NH_2_–Cit–Eu nanoprobe and its sensing mechanism for TC (Adopted by [39]).

Dye-doped UiO-66 was employed as a fluorescent reference, whereas a grafted Eu^3+^ complex functioned as the responsive signaling unit for tetracycline (TC) detection in food samples [39]. Upon increasing TC concentrations, the Eu^3+^-based fluorescence was selectively enhanced, producing a distinct ratiometric response accompanied by a visible color change from blue to red. The synthetic route for dye@UiO-66-@SiO_2_-NH_2_-Cit-Eu is illustrated in Figure 3.

The linearity was excellent over the concentration range of 0.1–6 µM. The LOD (17.9 nM) was adequate, being much lower than specification limits established by the U.S. Food and Drug Administration (FDA) (676 nM) and the European Union (225 nM). Satisfactory recoveries (97.05–106.01%) and good reproducibility (<4.91%). Furthermore, a low-cost paper-based probe was fabricated by immobilizing the nanoprobe onto filter paper, enabling rapid and visual detection of TC.

Another PAD approach has been reported for the analysis of sulfur dioxide in foods [40]. This method is based on headspace thin-film microextraction with smartphone-based on-cell detection. The method relies on the conversion of sulfite to sulfur dioxide through acidification of the sample solution. Headspace thin-film microextraction was conducted by exposing a cellulose paper strip, pre-impregnated with Fe(III), 1,10-phenanthroline, and acetate buffer (pH 5.5), to the sample headspace under controlled conditions. The sulfur dioxide absorbed onto the thin film reduced Fe(III) to Fe(II), leading to the formation of a red-colored Fe(II)–1,10-phenanthroline complex, which was subsequently detected directly on the paper substrate using a smartphone. A Samsung Galaxy J7 equipped with a 13 Megapixel camera was attached in front of a lab-made wooden box. The linearity of the method was studied in range of 0.1–700 ppb while high sensitivity (LOD = 0.04 ppb) was achieved. Adequate recoveries were recorded (94–103%).

A smart dual-readout sensing platform combining fluorescence and colorimetry was developed for the simple and rapid detection of *E. coli*, based on Cu^2+^-triggered oxidation of o-phenylenediamine [41]. In the absence of *E. coli*, Cu^2+^ oxidizes o-phenylenediamine to its oxidized form producing orange–yellow fluorescence along with a visible pale-yellow color. In contrast, the presence of *E. coli* effectively reduces Cu^2+^ to Cu^+^, thereby suppressing the Cu^2+^-mediated oxidation of o-phenylenediamine. A filter paper-based visual sensor was constructed and integrated with the OPD-Cu^2+^ system, with signal observation facilitated by UV illumination. The calibration curve was described by the equation *y* = 0.170 *x* + 0.300 with a correlation coefficient of *R*^2^ = 0.993, over an *E. coli* concentration range of 10^2^–10^6^ CFU/mL. By integrating the o-phenylenediamine-Cu^2+^-immobilized paper-based sensor with smartphone-based analysis, *E. coli* detection becomes simple and convenient, demonstrating strong potential for practical applications.

Rapid determination of nitroxynil in food using PAD has been proposed by Z. Xu et al. [42]. The target analyte is an anthelmintic veterinary drug that is widely used for the prevention and treatment of fascioliasis [43]. The principle of the method was based on the utilization of a novel flavone-based dye which binds to human serum albumin to form a dye-albumin complex that functions as a fluorescent probe for analyte detection. The fluorescence color changes of the solution under 365 nm UV irradiation were readily visible to the naked eye. The specificity of the method was assessed in the presence of commonly used anthelmintic drugs. According to the authors, the analysis time was very fast (≤5 s) with high sensitivity (LOD ∼ 107 nM).

**Figure 4:**
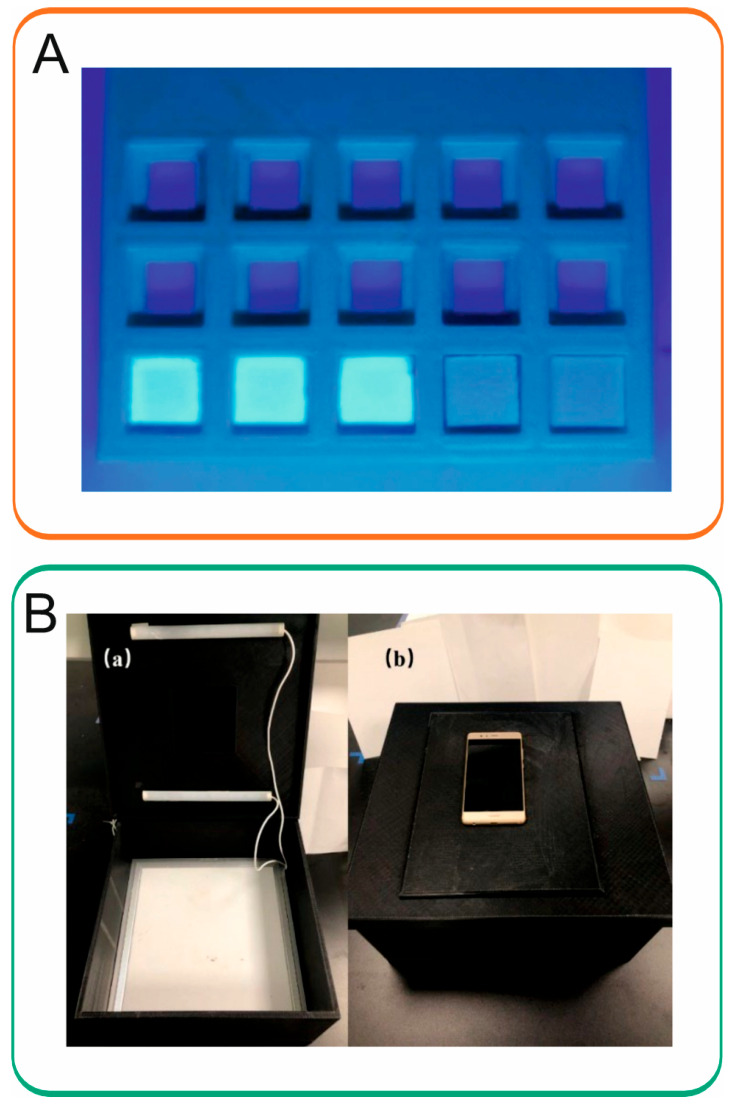
(**A**) Mold incorporating CQD paper and plain paper, (**B**) 3D-printed color detection box, (**a**) internal view, and (**b**) assembled with a smartphone (adopted from [44]).

In 2022, an online reactor was constructed for the fabrication of nitrogen-doped carbon quantum dots (CQDs) [44]. The optical properties of the CQDs were systematically characterized. The CQDs exhibited fluorescence quenching in the presence of common phenolic compounds and this principle was employed for the determination of total phenolic content in honeysuckle extracts. Two UV lamps inside were used as the excitation light sources (365 nm) and Color Grab version 3.3.0 was utilized for color analysis. The mold CQD paper and the 3D-printed color detection box are shown Figure 4. The method was linear in the range of 0.2–1.6 mg/mL with an LOD of 0.271 mg/mL. The analysis revealed that honeysuckle extracts contained total phenolic content ranging between 0.885 and 1.60 mg/mL, corresponding to 1.77–3.20% per unit mass of herb after conversion. Notable variations in total phenolic content were observed among different batches of honeysuckle water extracts.

The fluorescence characteristics of flavonoids were investigated in aqueous to borax-containing solutions [45]. The reported results showed that the inherently weak fluorescence of flavonols can be markedly enhanced in the presence of borax. The natural flavonol morin was selected as a model fluorescent probe to construct a turn-on sensing strategy for borax detection. The fluorimetric method exhibited a linear response over four orders of magnitude, with an LOD of 1.07 µM. In parallel, a smartphone-assisted PAD was fabricated using 3D-printing technology. Morin-impregnated paper strips enabled visual detection of borax through RGB analysis of captured images, achieving an LOD of 0.13 mM (equivalent to 27.05 µg mL^−1^ as Na_2_B_4_O_7_). Both approaches were successfully applied to the analysis of various real samples, yielding recoveries of 86.9–106.3% for batch fluorimetry and 82.7–108.8% for smartphone-assisted fluorescence sensing.

Quantitation of doxorubicin at trace levels was performed using a PAD [46]. The LOD was 83 nM, using doxycycline visual sensing via interaction with BSA on the surface of red-emitting copper nanoclusters. The drug bound to the hydrophobic cavities of albumin, where the nonpolar microenvironment restricted solvent interactions and reduced nonradiative decay pathways. As a result, the fluorescence intensity of doxorubicin was significantly enhanced. Protein precipitation using 10% trifluoroacetic acid was employed for milk sample cleanup. The recovery rates for doxorubicin ranged from 96.4% to 105%, with RSD values below 3.54%.

In 2024, the research group of I. Ortiz-Gomez reported the development of a microfluidic PAD for the fluorimetric determination of *β*-lactoglobulin [47]. A *β*-lactoglobulin-specific aptamer was immobilized onto a cellulose disk to act as the recognition element, while fluorescent CQDs were bio-conjugated with a complementary oligonucleotide to function as a signal-labeling probe through aptamer–complementary DNA hybridization. The formation of the CQD-single-stranded DNA conjugates was verified by two independent agarose gel electrophoresis assays, confirming successful bioconjugation. The method exhibited excellent sensitivity toward *β*-lactoglobulin with a linear detection range of 1.8–500 ng/mL and an LOD of 0.6 ng/mL. In addition, the proposed method was successfully validated for the quantification of *β*-lactoglobulin in diverse food matrices, including skimmed milk, crispy rice, cookies, and chocolate, demonstrating satisfactory analytical performance across all tested samples.

An enzyme-free fluorimetric strategy was developed for the sensitive and selective detection of *o*,*o*-dimethyl-*o*-2,2-dichlorovinyl phosphate, exploiting the excellent peroxidase-like activity of CuS nanoparticles [48]. The assay operated under H_2_O_2_-free and enzyme-free conditions, which significantly minimizes environmental interference and enhances reliability. In addition, the method was successfully applied to the analysis of real samples, yielding satisfactory recoveries ranging from 86% to 106%, thereby demonstrating its practical applicability. Owing to its simple preparation and straightforward operation, the assay is well suited for integration into paper-based test strips. The sensing system exhibited excellent specificity, responding exclusively to the analyte without interferences. A linear range between 0.0001 and 0.1 µg/mL with an LOD of 0.1 ng/mL was achieved. Moreover, the authors fabricated paper-based test strips for visual detection of the analyte under UV irradiation. When combined with a smartphone equipped with a Color Picker application, the platform enabled POC testing with quantitative capability.

**Figure 5:**
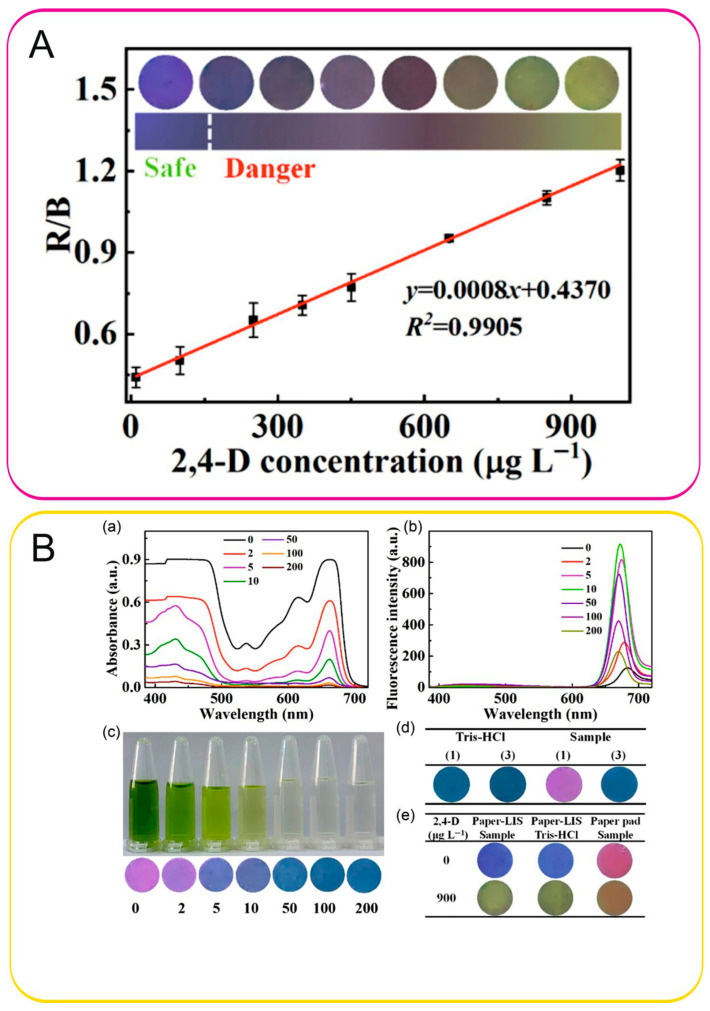
(**A**) The calibration curve of 2,4-dichlorophenoxyacetic acid. (**B**) UV–vis (**a**) and fluorescent spectra (**b**) of extract solution of Chinese cabbage without 2,4-dichlorophenoxyacetic acid treatment at different dilution times: 0–200; (**c**) images of extract solution of Chinese cabbage with different dilution times using a UV lamp (@365 nm); (**d**) images of filter holders with the addition of Tris buffer; (**e**) photographs for analysis of the analyte in extract solution (adopted from [49]).

In 2023, researchers developed a lab-in-a-syringe device incorporating a multi-emitting fluorescent system for the on-site determination of 2,4-dichlorophenoxyacetic acid [49]. The approach was based on the specific inhibition of alkaline phosphatase activity by the analyte, thereby suppressing the generation of ascorbic acid. MnO_2_ nanosheets oxidize the ascorbic acid to dehydroascorbic acid, which subsequently reacts with *o*-phenylenediamine to produce a blue-emitting product at 435 nm. Meanwhile, the remaining MnO_2_ nanosheets further react with *o*-phenylenediamine to form a yellow-emitting product (λ_em_ = 570 nm). A linear calibration curve was obtained in the range of 10–1000 μg/L (Figure 5A). Sensitive and visual detection of 2,4-D with an LOD of 5.0 μg/L, recoveries of 94.6–106.8%, and RSDs of 2.3–6.8% was achieved. The method was applied to the analysis of Chinese cabbage and carrot samples (Figure 5B).

A simple fluorometric PAD sensor was proposed for the quantitation of quinine [50]. Without involving any chemical reaction, the method uses quinine’s inherent fluorescence after pH regulation with nitric acid, enabling room-temperature detection on a paper platform under 365 nm UV irradiation. The authors found that a concentration of 0.1 M HNO_3_ and a reaction time of 10 min were required to obtain high fluorescence intensity. The method exhibited a limit of detection (LOD) of 3.6 ppm, which is acceptable for this application but does not represent a significant improvement over existing fluorimetric approaches. The method was applied to the analysis of tonic water, and the results showed reasonable agreement with those obtained using a conventional batch spectrofluorimetric method, although no clear advantage in sensitivity was observed.

A microfluidic PAD coupled with MIP technology was used for the determination of histamine in canned tuna [51]. Histamine, a naturally occurring biogenic amine, is a key mediator in physiological processes such as inflammation and allergic reactions. Differences in the structure of histamine receptors on cell membranes contribute to the wide variability in histamine responses among individuals [52]. MIPs were synthesized by precipitation polymerization and employed as dispersive solid-phase extraction sorbents for the selective extraction of the analyte. A laborious pretreatment protocol of tuna samples followed involving homogenization, extraction with mixture water/acetonitrile (40/60 *v*/*v*), centrifugation and liquid extraction with hexane prior to dispersive solid-phase extraction. A linear correlation was observed between the color ratio and histamine concentration over the range of 10–100 ppm. The LOD calculated at a signal-to-noise ratio was found to be 7.99 ppm.

**Table 1:** Applications of PAD-based methods in food analysis.

Analyte	Sample	Method Principle	λ_ex_ (nm)	LOD	Color Processing Application	Ref
TC	Foodstuffs	Ratiometric fluorescence using dye@UiO-66 as internal reference and Eu^3+^ complex as signal unit; TC enhances Eu^3+^ fluorescence (blue → red)	–	17.9 nM	RGB analysis by smartphone	[39]
Sulfite	Apple cider vinegar, lime juice, sour orange juice	Acidification converts sulfite to SO_2_; SO_2_ reduces Fe(III) to Fe(II) forming red Fe(II)–phenanthroline complex	–	0.04 ppb	RGB analyzer software for Android	[40]
*E. coli*	Degrease milk, tap water	Cu^2+^-OPD system; bacteria reduce Cu^2+^ suppressing oxidation of OPD; fluorescence & colorimetry dual-readout	414	44 CFU/mL, 100 CFU/mL	Smartphone-assisted RGB analysis	[41]
Nitroxynil	Milk, Beef	Flavone dye–albumin fluorescent probe; visible fluorescence color change	410	107 nM	ColorDesk Pro, ver 1.2.5	[42]
Total phenolic compounds	Honeysuckle extracts	CQDs fluorescence quenching by phenolics	365	0.213–0.287 mg/mL	Color Grab app (v3.3.0)	[44]
Borax	Bean, flour, meat	Morin fluorescence “turn-on” in presence of borax	460	1.07 µM (fluorimetry); 0.13 mM (paper)	ImageJ	[45]
Doxorubicin	Milk	Interaction with BSA–Cu nanoclusters enhancing fluorescence	365	45 nM	Color Recognizer APP	[46]
β-Lactoglobulin	Milk, cookies, chocolate, rice	Aptamer–CQD fluorescent probe in microfluidic PAD	365	0.6 ng/mL	ArtCAM JewelSmith 2011	[47]
o,o-Dimethyl-o-2,2-dichlorovinyl phosphate	Apple, tomato	CuS nanoparticles with peroxidase-like activity, fluorimetric sensing	540	0.1 ng/mL	Color Picker app	[48]
2,4-D (2,4-dichlorophenoxyacetic acid)	Vegetables	Multi-emission fluorescence system based on enzyme inhibition and OPD reactions	365	5.0 µg/L	Color recognition	[49]
Quinine	Tonic water	Native fluorescence of quinine on PAD after pH adjustment	360	3.6 mg/L	ImageJ	[50]
Histamine	Canned tuna	MIP extraction + microfluidic PAD, color ratio measurement	360	7.99 ppm	Color Analyzer	[51]
Sulfamethazine, oxytetracycline, chloramphenicol	Shrimp	Aptamer–CDs/MoS_2_ FRET “off–on” fluorescence	365	0.34–0.48 ng/mL	-	[53]
Methiocarb	Cauliflower, cabbage	Reaction with nitrogen-doped carbon quantum dot (N-CQDs)	450	0.35 ng/mL	ImageJ	[54]
Iodate	Food/water	Fluorescence quenching of BSA–Au nanoclusters with gas separation PAD	490	0.005 mM (fluorimetric), 0.01 mM (image)	ImageJ	[55]
Oxytetracycline	Milk	Eu(III)/CDs ratiometric fluorescence on paper	310	25 nM	Maarten Zonneveld app	[56]
Cu^2+^	Sugar cane spirits	Rice-derived CDs functionalized with cuprizone	370	0.23 ppm	Color name app	[57]
Folic acid	Celery, Broad bean, lettuce, citrus, pear, kiwi, yolk	Reaction with gadolinium and nitrogen co-doped CDs	340	3.43 nM	Color Coll app	[58]
β-Lactoglobulin	Milk, cookies, rice porridge	QD–aptamer–graphene oxide coffee-ring fluorescence quenching	–	48 ppb	ImageJ	[59]
Benzoyl peroxide	Wheat flour, noodles, baking powder	Oxidation of phenothiazine dye (red → yellow), fluorescence turn-on	454	257 nM	Color Picker	[60]
Fluoroquinolones	Milk	Eu-MOF fluorescence & color change (IFE + PET mechanisms)	360	9.3–238.6 nM	-	[61]
Pd^2+^	Chinese mitten crab, Silver carp, Whiteleg shrimp, Procambarus clarkii, squid, Cuttlefish balls	Aptamer–ZnSe@ZnS QD fluorescence quenching	386	0.56 nM	ImageJ	[62]
Aflatoxin B1	corn, rice and peanut	Dye@MOF turn-on ratiometric fluorescence	330	4.16 nM	-	[63]
Glyphosate, malathion, acetamiprid	Matcha powder, black tea,	Multichannel PAD biosensor with cloud & smartphone imaging	980	0.285–0.493 ng/mL	Smartphone + cloud platform	[64]
Glutathione	Nutritional supplements	Direct fluorimetry on PAD after OPD derivatization	365	20.5 μM	ImageJ	[65]
Glutathione	Dietary supplements	Reaction with o-phthaldialdehyde functionalized silica particles	340	0.34 μM	-	[66]
Sodium hypochlorite, hydrogen peroxide and formaldehyde	Milk	Reaction with 8 different nanoclusters	280–360	0.01–0.05%	ImageJ	[67]
Quercetin	Orange juice, tea	Fluorimetric detection after magnetic MIP extraction	430	1.1 ng/mL	-	[68]

A rapid on-site detection platform based on a laser-printed paper microfluidic chip incorporating multicolor fluorescent nanoprobes was developed for the analysis of sulfamethazine, oxytetracycline, and chloramphenicol. While the platform enabled simultaneous detection through distinguishable fluorescence responses and offered the advantage of portability, its analytical performance was primarily demonstrated under controlled conditions, and comprehensive evaluation in complex real food matrices was limited [53]. The “fluorescence-off” sensing probes consisted of CDs conjugated with aptamers as energy donors and MoS_2_ nanosheets as acceptors, operating via a Förster resonance energy transfer mechanism. Under optimized conditions, the PAD achieved a rapid response time of 15 min with high sensitivities of 0.47, 0.48, and 0.34 ng/mL for sulfamethazine, oxytetracycline, and chloramphenicol, respectively. When applied to shrimp samples, good recoveries ranged from 95.2 to 101.2%, 96.4 to 105%, and 96.7 to 106.1%, with RSDs below 6%. This paper-based sensing platform provided a promising route for high-throughput, rapid, and on-site detection, with broad potential for POC testing in food safety applications.

In 2023, Patel, S. et al. proposed a portable smartphone-assisted digital image fluorimetry for analysis of methiocarb pesticide in vegetables [54]. Fluorescent paper-based detection was conducted by depositing methiocarb onto paper substrates functionalized with N-doped CQDs. The resulting fluorescence changes were captured using a smartphone camera under controlled illumination conditions and subsequently quantified using ImageJ software. Am adequate linear response was obtained for methiocarb analysis in the range of 10–1000 µg/L with a low LOD of 3.5 µg/L using conventional fluorimetry, and 700–10,000 µg/L with an LOD of 500 µg/L using the fluorescent paper sensor. Recoveries ranging from 92.0 to 95.4% demonstrated the good selectivity of both methods for methiocarb determination in vegetable samples. Overall, the use of *N*-doped CQDs as fluorescent sensors offers an instrument-free, portable, and user-friendly approach for methiocarb analysis in vegetables.

A. Lert-Itthiporn introduced a foldable PAD for iodate determination [55]. This system operates through membraneless gas separation and fluorescence quenching of gold nanoclusters. A rectangular PAD was fabricated in a folded configuration by patterning two circular reservoirs, designated as the donor and acceptor reservoirs, onto a single paper substrate, allowing the reservoirs to be aligned upon folding for straightforward operation. Fluorescence quenching of bovine serum albumin (BSA)-protected gold nanoclusters constituted the detection mechanism. The nanocluster solution was first introduced into the acceptor reservoir, while the sample solution, iodide, and sulfuric acid were subsequently and sequentially added to the donor reservoir to initiate the sensing process. After folding the PAD, the donor and acceptor reservoirs were brought into contact and secured together using double-sided mounting tape. Two calibration curves were constructed for the respective detection modes, both exhibiting good linearity (*r*^2^ > 0.98) over the iodate concentration range of 0.005–0.1 mM. The method demonstrated high accuracy, with a mean recovery of 95.1 ± 4.6%, and excellent precision, as indicated by RSDs below 3%. The LODs were 0.005 mM for fluorometric detection and 0.01 mM for image-based analysis.

An innovative fluorescent Eu(III)-based ratiometric probe was developed for the determination of oxytetracycline in milk [56]. The sensing system relies on adenosine monophosphate (AMP)/Eu(III) nanoscale coordination polymers, formed via self-assembly of Eu^3+^ and AMP on the surface of carbon dots enriched with hydroxyl and carbonyl functionalities. The doped NCPs were uniformly immobilized onto conventional filter paper to fabricate a visual ratiometric probe. With the assistance of a digital camera equipped with a color-detection application (Maarten Zonneveld), the paper-based test strip enabled quantitative determination of the analyte, achieving an LOD of 0.5 µM and a wide linear range of 1–100 µM. A quite similar approach has been followed by the research group of W. T. Suarez for the determination of Cu^2+^ in sugar cane spirits [57]. A UV-LED chamber and a 3D platform fabricated from biodegradable polylactic acid were employed in combination with rice-derived CDs functionalized with cuprizone. Four UV-LEDs with a peak emission wavelength of 370 nm were installed in a chamber to serve as the excitation source. A linear response was obtained over the concentration range of 2.00–7.22 mg/L using the blue channel, with an LOD of 0.23 ppm and an excellent correlation coefficient (*R*^2^ = 0.9993). Recoveries ranging from 87.60 to 112.4% indicated that no significant matrix effects were present. L. Liu took advantage of using gadolinium and nitrogen co-doped CDs for the quantitation of folic acid in vegetable, fruits, and yolk samples [58]. Gd,N-doped CDs (Gd,N-CDs) were synthesized via hydrothermal treatment of Gd(NO_3_)_3_, o-phenylenediamine, and 4-aminobenzoic acid. Upon addition of folic acid, the emission intensity of the Gd,N-CDs at 560 nm decreased, accompanied by a corresponding increase in fluorescence intensity at 457 nm. Quantitative determination of folic acid was therefore achieved using the fluorescence intensity ratio *F*_457_/*F*_560_.

An interesting approach based-on coffee-ring effect paper sensor chip for the determination of *β*-lactoglobulin in foods via a smartphone has been proposed [59]. The sensing strategy relied on a quantum dot–aptamer–graphene oxide assembly, in which graphene oxide efficiently quenched the fluorescence of the quantum dots through close-range interactions. Upon binding of β-lactoglobulin to the aptamer, the assembly was disrupted, leading to a reduction in quenching efficiency; consequently, the fluorescence intensity increased in proportion to the β-lactoglobulin concentration. For signal quantification, images of the fluorescent coffee-ring patterns were captured using a smartphone and analyzed with self-developed software. Quantitative determination of *β*-lactoglobulin was rapidly achieved based on the intensity of the resulting fluorescent coffee ring. The method provided an LOD of 0.048 ppm and a linear working range of 0.39–1000 ppm. The assay was successfully validated using real food samples, with the obtained results showing good agreement with those generated by a commercial ELISA kit, thereby confirming its analytical accuracy and practical applicability.

A turn-on probe based on the usage of (*E*)-2-(3-(2-(10-ethyl-10H-phenothiazin-3-yl)vinyl)-5,5-dimethylcyclohex-2-en-1-ylidene)malononitrile for the determination of benzoyl peroxide has been reported [60]. The electron-donating sulfur atom in the phenothiazine moiety can be directly oxidized by the analyte to an electron-withdrawing sulfoxide (S=O) group, leading to a pronounced color change from red to yellow accompanied by a simultaneous fluorescence turn-on response. The smartphone-based sensing platform was applied to the determination of the analyte in wheat flour, noodles, and baking powder, achieving high accuracy (91.35–95.74%), excellent sensitivity, and an LOD of 257 nM.

A cage-based lanthanide MOF was fabricated for the determination of fluoroquinolones in foodstuffs [61]. A three-cage lanthanide MOF (1-Eu), was constructed using a C_2_-symmetric 5,5′-(pyrazine-2,6-diyl)diisophthalic acid ligand, with Eu^3+^ clusters serving as the structural building units. This probe enabled the detection of four fluoroquinolone antibiotics with low LODs (9.3–238.6 nM). However, while the sensitivity is noteworthy, the detection performance varied substantially among the analytes, and the practical applicability in complex matrices may be constrained by the synthetic complexity and structural specificity of the MOF. Mechanistic investigations indicated that the sensing behavior was governed primarily by internal filter effects and photoinduced electron transfer processes. The latter mechanism involves a process in which light absorption triggers the transfer of an electron from a donor molecule to an acceptor molecule.

Ultrasensitive detection of Pd was carried out using a smartphone-aided paper imprinted aptasensor [62]. The sensing system utilized ZnSe@ZnS quantum dots as the fluorescence reporters and aptamers as the specific recognition probes. A Box–Behnken design was used for the optimization of the method’s parameters through 17 experimental runs. The fluorescence quenching value increased from 60 to 130 as the salt ion concentration increased and subsequently decreased to 90 at higher levels. Spiked recovery experiments conducted on six aquatic product matrices, including prawns, crabs, and fish, yielded recoveries in the range of 88.06–107.29%. The proposed biosensor achieved a rapid detection time of 1 min with a limit of detection of 47.73 µg kg^−1^. While the short analysis time is advantageous for on-site screening, the sensitivity may be insufficient for trace-level detection required in certain food safety and environmental monitoring applications.

In 2025, Li, Z. and co-workers introduced a Dye@MOFs-based turn-on ratiometric fluorescence system enabling smartphone-assisted visual sensing of aflatoxin B1 on paper. [63]. The grain samples were first mixed with water/methanol 3/7 *v*/*v*, homogenized and centrifuged. The optical properties of the material were investigated through UV–vis absorption and fluorescence measurements. The method achieved an LOD of 29.89 nM over a concentration range of 0.04–12.8 µM, accompanied by a visible color transition from light pink to blue under UV illumination, enabling a simple and effective approach for on-site determination of the aflatoxin B_1_.

Simultaneous determination of glyphosate, malathion, and acetamiprid was achieved using a multichannel microfluidic PAD biosensor integrated with a cloud platform [64]. The proposed method demonstrated a rapid response time of 25 min for glycine, malonic acid and acetic acid achieving fluorescence-based LODs of 0.133, 0.181, and 0.232 ppb, respectively, and image-based LODs of 0.493, 0.346, and 0.285 ppb, respectively. No significant differences were observed between this method and the HPLC reference method.

A novel direct PAD approach was reported for the analysis of glutathione in nutritional supplements [65]. After irradiation at 365 nm, the fluorescence intensity emitted from the surface of the PAD was proportional to the glutathione concentration and was quantified using a smartphone as the detector. The authors found that derivatization of the analyte with *o*-phthalaldehyde is favored at pH 11, consistent with previous studies that identified the optimal pH range to be between 10 and 12 [66]. The minimum time required to obtain measurable results was 7 min, which is acceptable but not particularly rapid compared with other reported sensing approaches. The intra-day and inter-day RSDs were less than 7.3% indicate acceptable repeatability rather than high analytical precision.

A set of 8 nanoclusters was synthesized and used to evaluate milk adulteration [67]. The technique was further extended to detect and quantify sodium hypochlorite, hydrogen peroxide, and formaldehyde as adulterants. The investigated adulterants can influence the fluorescence behavior of the nanocluster-based sensor array through two main mechanisms. First, they may indirectly interact with milk constituents by reacting with small metabolites or proteins, thereby perturbing the equilibrium between these components and the nanoclusters and altering the local microenvironment of the nanoclusters. Second, they may directly engage with the nanoclusters via redox reactions, inducing changes in the electronic structure and surface states of the nanoclusters, which ultimately affects their fluorescence behavior.

Selective extraction and quantification of quercetin in plant matrices were achieved using a newly developed magnetic MIP [68]. The material was characterized using FTIR, X-ray diffraction, SEM, TEM, and thermogravimetric analysis, confirming its successful fabrication. The fabricated magnetic MIP was employed as a selective sorbent in solid-phase extraction for sample preparation. In addition, a sensitive fluorometric method was developed for the quantification of quercetin, exhibiting a linear response over the concentration range of 0.005–1.25 µg/mL. The LOD and LOQ were 1.1 and 3.7 ng/mL, respectively. In spiked orange juice and tea extract samples, average recoveries for quercetin ranged from 92.2 to 104.7%, with RSDs below 5.06%.

In summary, PAD-based approaches offer promising tools for rapid food safety screening, though issues such as matrix interference, assay reproducibility, and regulatory validation continue to limit large-scale implementation.

### 3.3. Environmental Analysis

PADs are commonly used for the detection and monitoring of environmental contaminants in water, soil, and air samples. The paper substrate (typically cellulose filter paper) serves multiple functions: it acts as a support for reagents, a medium for fluid transport via capillary action, and a platform for chemical reactions. Samples are applied directly to the paper, eliminating the need for external pumps or complex instrumentation. The application of PADs in environmental analysis is summarized in Table 2.

A novel reaction-based fluorometric probe was developed for the monitoring and quantification of peracetic acid, in which peracetic acid induces oxidative hydroxylation of the phenylboronic acid moiety within the dye molecule. This chemical transformation alters the electronic structure of the fluorophore, resulting in a measurable change in fluorescence that enables quantitative detection [69]. The probe displayed strong and selective fluorescence responses toward the analyte compared with other commonly used oxidants, as well as typical metal ions and anions. The probe functioned efficiently across a broad pH range (4.0–10.5) and achieved an LOD of 4.6 × 10^−8^ M (3.5 ppb). In addition, analyte’s levels could be conveniently quantified using a smartphone by analyzing changes in the blue-channel intensity of captured images. Under 365 nm UV irradiation, solution images were recorded in a dark room using an iPhone X, and the corresponding RGB values were obtained with a color analysis application (White Marten, ColorMeter RGB Hex color picker and colorimeter). Finally, successful quantitative detection of airborne peracetic acid was demonstrated using paper-based strips impregnated with the probe.

Fluorescent CQDs were prepared via solvothermal treatment of orange peel for the determination of permanganate [70]. The resulting CQDs were relatively well dispersed, with an average particle size of 3.14 ± 0.71 nm. The CQDs exhibited excitation-dependent fluorescence behavior, with a λ_ext._/λ_em._ 400/506 nm. However, this behavior is common among CQDs and does not by itself indicate superior optical performance or uniform surface states. Upon the addition of permanganate ions, the fluorescence intensity of the CQDs decreased progressively due to the combined effects of the inner filter effect (IFE) and static quenching effect. These findings demonstrate that the CQDs can function as an effective fluorescent probe with high sensitivity for the quantitative detection of permanganate, achieving a low LOD of 3.31 µM. An almost similar fluorescent probe was proposed by Fu, W. et al. for the sequential analysis of Fe^3+^ and ascorbic acid in environmental water samples [81]. The determination of Fe^3+^ exhibited a linear response over the concentration range of 2–150 µM, with an LOD of 0.253 µM. In addition, within the ascorbic acid concentration range of 30–130 µM, the recovered fluorescence showed good linearity yielding a calculated LOD of 1.57 µM.

A selective and sensitive optical probe for the ratiometric detection of Fe(III) and Cu(II) in solution was developed [71]. The method is based on the formation of an *N*-linked disalicylaldehyde derivative. The probe exhibited rapid fluorescence turn-off behavior accompanied by visible color changes within 60 s. Sequential titration experiments revealed coordination stoichiometries of 1:1 for the H_2_ disalicylaldehyde-Fe(III) complex and 2:1 for the H_2_ disalicylaldehyde-Cu(II) complex. The corresponding limits of detection were determined to be 0.40 µM for Fe(III) and 0.21 µM for Cu(II). In addition, a disalicylaldehyde-coated PAD was developed, offering a simple platform for the detection of Fe(III) and Cu(II). The recoveries from the analysis of water samples ranged between 99–103%.

**Figure 6:**
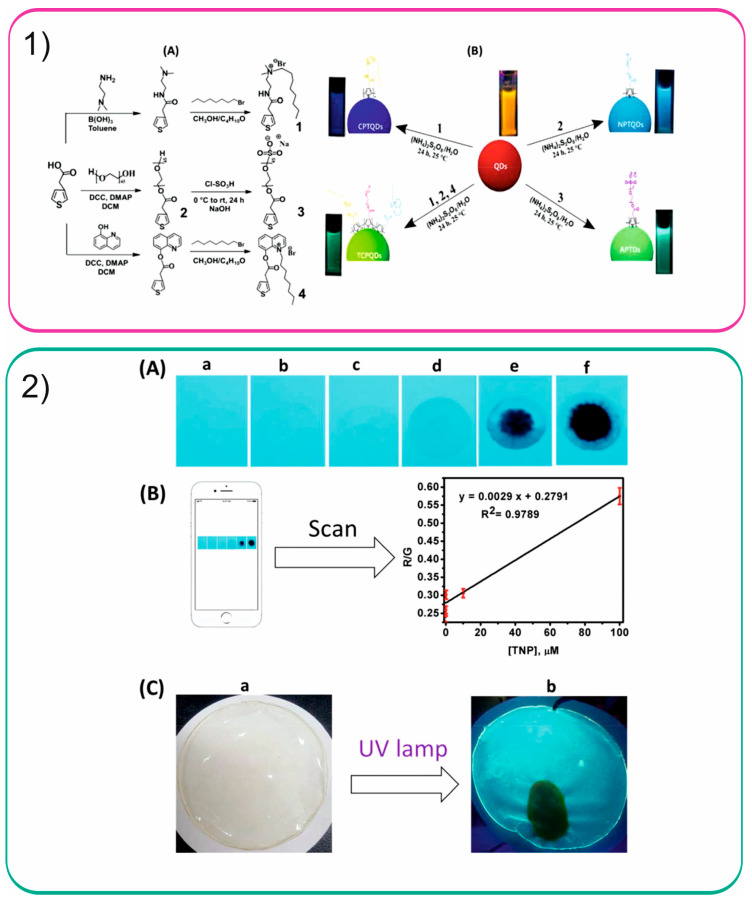
(**1**) (**A**) A reaction scheme illustrating the synthesis of various amphiphilic thiophene monomers and (**B**) the fabrication of multicolor-emissive amphiphilic conjugated polythiophene–coated CdTe QDs via an in situ polymerization approach, (**2**) (**A**) Fluorescence images before and after the addition of 10 µL of 2,4,6-trinitrophenol solutions at different concentrations: water 10^−12^–10^−4^ M; (**B**) calibration curve; (**C**) thiophene copolymer nanohybrid-doped transparent chitosan film under daylight (**a**), and its fluorescence image under UV light (adopted from [72]).

Unique polythiophene-coated QDs with multiple emission characteristics were developed for the determination of the explosive 2,4,6-trinitrophenol [72]. Four biocompatible sensing systems were fabricated via an in-situ polymerization approach, resulting in markedly enhanced fluorescence intensity and quantum yields of up to 78%. The reaction schemes are shown in Figure 6. The fluorescence quenching efficiency of the analyte toward the synthesized QDs reached a maximum at pH 7.0, likely due to the strong binding affinity between the sensor sites and 2,4,6-trinitrophenol under neutral conditions. This finding indicates that the sensors are well suited for application in real water samples, as environmental waters typically exhibit near-neutral pH. A linear relationship for the analyte in the range of 1–120 × 10^−9^ M with correlation coefficient higher than 0.9903 in all fabricated QDs. Adequate recoveries were obtained for the analysis of tap and river water samples ranged between 98.02 and 106.75%.

A low-cost instrumental-free approach for bromine speciation was developed using 5-oxo-3,5-dihydro-2 H-thiazolo[3,2-a]pyridine-7-carboxylic acid (TPCA) [73]. Bromine was generated in situ and simultaneously captured and reacted at the detection zone of a TPCA-modified PAD, with detection performed via a smartphone. A Plackett–Burman experimental design was applied to identify the most influential parameters. It was noted that increasing the H_2_SO_4_ concentration significantly enhanced the analytical signal, since sulfuric acid is essential for effective in situ bromine vapor generation. Under optimized conditions, the proposed method achieved limits of detection of 5.4 µg/L for Br^−^ and 0.9 µg/L for BrO_3_^−^, with repeatability better than 10% for both bromine species.

The quantitation of copper in environmental water samples was performed though its catalytic reaction with azide-alkyne cycloaddition reaction [74]. The concentration of Cu(II) was quantified by RGB analysis using an iPhone application. Owing to the strong catalytic activity of CLICK-T toward the Cu(II)-catalyzed azide–alkyne cycloaddition reaction, a naked-eye detection limit of 0.1 µM was achieved. Acceptable recoveries in the range of 89–102% were achieved, with RSDs between 1.3% and 5.8%. Compared with conventional laboratory analyses, this approach substantially simplifies the detection procedure, reduces analysis time, and demonstrates strong practical applicability. More recently, blue and yellow fluorescence CQDs were synthesized for the determination of Cu^2+^ in tap water and rice samples [83]. The dual-emission ratiometric fluorescence sensor (B@Y-CQDs), prepared via a physical blending approach, enables dual-mode detection of copper ions, offering both colorimetric (visual) and ratiometric (quantitative) sensing capabilities. An LOD of 0.73 μM within the linear range of 7–15 μM was achieved.

A CD-based FRET system was constructed using CDs and Ru^3+^ complexes as the energy donor–acceptor pair for the visual detection and efficient removal of Hg^2+^ [75]. FRET occurs when the emission spectrum of a fluorescent donor overlaps with the absorption spectrum of an acceptor [31]. The presence of Hg^2+^ induced a pronounced linear increase in the ratiometric fluorescence signal, achieving an LOD of 95 nM, accompanied by continuous fluorescence color changes from blue to red. A color transition of the test paper was readily observed by the naked eye using UV radiation at 365 nm. After spiking tap water and wastewater samples with different concentrations of Hg^2+^ (0.01–10.00 µM), recoveries in the range of 93.00–107.70% were obtained, with RSD values below 5.00% for three replicate measurements, demonstrating the high reliability and practicality of the proposed method. Using carbon dots and fluorescence digital imaging, the research group of W. T. Suarez determined Hg^2+^ in water samples [77]. The synthesis was economical, using rice starch. The particle sizes of CQs were between 0.5 and 3 nm, with an average size of ca 1 nm. Functionalization of the CDs with methimazole imparted high selectivity toward Hg^2+^ detection. The analytical response was linear over the concentration range of 0.5–45.0 µmol/L, with R^2^ of 0.997. The LOD and LOQ were 0.23 and 0.62 µM, respectively. Statistical comparison at the 95% confidence level indicated no significant differences between the results obtained using the proposed method and those from the reference method. The quantitative detection of Hg^2+^ was reported after its complexation with NaGdF_4_:Eu/CDs nanocomposites [86]. The excitation spectrum displayed a pronounced peak at 274 nm and a weaker peak at 395 nm. When excited at 274 nm, NaGdF_4_:Eu displayed characteristic Eu^3+^ emission bands at 596 nm and 621 nm. Compared to method [77] lower LOD (20.8 nM) was achieved. For comparison, the concentration of Hg^2+^ in spiked samples was determined using inductively coupled plasma optical emission spectrometry (ICP-OES). The recoveries ranged from 95.9% to 101.6%, with RSDs between 0.16 and 2.4%. A simple and ultrasensitive paper-based visual fluorescent chip was developed for detecting trace Hg ions in environmental water [87]. CdTe quantum dots anchored on silica nanospheres were immobilized on paper fibers to prevent fluorescence unevenness caused by liquid evaporation, enabling stable and uniform sensing. Hg ions selectively quenched the quantum dot fluorescence at 525 nm, and the visual results were captured using a smartphone. The method achieved a low detection limit of 2.83 µg/L with a fast response time of 90 s and showed high accuracy in various real water samples with recoveries of 96.8–105.4%. This low-cost, user-friendly approach shows strong potential for commercial application and large-scale environmental monitoring.

In 2022, Chen, X. et al. proposed a bifunctional fluorescent nanoprobe for the determination of dipicolinic acid (anthrax biomarker) and Cu^2+^ in water samples [76]. The ATP@Eu:Tb-MOF nanoprobe was prepared at ambient conditions using a simple one-step assembly of inorganic nanoclay ATP and lanthanide MOFs. Fluorescence imaging was performed by exposing the ATP@Eu:Tb-MOF nanoprobe to varying concentrations of DPA and Cu^2+^, followed by irradiation at 254 nm. At analyte concentrations between 2.5 and 8 μM, simultaneous green and red emissions occurred, resulting in a combined orange fluorescence. The fluorescence intensities of the nanoprobe at 545 nm and 617 nm gradually decreased with increasing Cu^2+^ concentration. The nanoprobe interacted with Cu^2+^, resulting in efficient quenching of the red fluorescence and achieving an LOD of 6.94 nM.

A similar approach to [46] has been proposed by Li, T. et al. for the quantitation of doxorubicin in environmental water samples [88]. The proposed strategy was based on the fabrication of red beetroot-derived carbon dots which were reacted with the analyte. CDs derived from red beetroot through a straightforward hydrothermal method displayed a cyan emission centered at 496 nm when excited at 416 nm. The analytical procedure involved the filtration of 100 mL water sample was followed by pH adjustment to 6.5–7.5 and the introduction of CDs. A variety of metals (K^+^, Na^+^, Ag^+^, Hg^2+^, Cu^2+^, Mg^2+^, Cd^2+^, Pb^2+^, Fe^2+^ and Al^3+^), amino acids (Cys, Ala, Pro, Ser, Asn, Trp and Leu) and medicinal excipients (Cac, Sta, Sur and Glu) were investigated in terms of selectivity. The LOD was calculated to be 78.3 nM within a linear concentration range of 3–35 µM.

Fluorine-free synthesis of Ti_3_C_2_ MXene quantum dots was proposed for fluorimetric determination of acetylcholinesterase and organophosphorus pesticides in soil samples [89]. Acetylcholinesterase-mediated hydrolysis of acetylthiocholine was first carried out to generate thiocholine. The resulting thiocholine was then allowed to react with Ehrman’s reagent, leading to cleavage of the reagent and formation of the yellow 2-nitro-5-thiobenzoate anion. Acetylcholinesterase catalyzed the hydrolysis of the substrate to produce 2-nitro-5-thiobenzoate anion, which efficiently quenched the fluorescence of Ti_3_C_2_ MXene quantum dots via the inner filter effect. The material was characterized using FTIR and XPS. To obtain linearity, a logarithmic transformation was followed in the range of 5–100 ppb with an LOD of 0.2 ppb. The selectivity of the method was satisfactory since the spiked samples provided acceptable recoveries in the range of 95.7–106.8%.

A dual-emissive Eu^3+^-based metal–organic framework (Eu^3+^-MOF) was developed for the determination of malachite green and leuco-malachite green in fishpond water samples, providing distinct emission responses that enabled sensitive and selective detection of both analytes [78]. The material was prepared by sequential pre-functionalization with a blue-emissive ligand and post-functionalization with red-emissive Eu^3+^ ions on a UiO-66 framework. The adsorption capacity toward the analyte rose sharply during the first 90 min and then increased more slowly, reaching equilibrium after 120 min. Furthermore, the prepared material showed high reusability and recycling capability in both analyte detection and removal processes, consistent with the concepts of green and sustainable chemistry [90]. The adsorption process was based on π–π stacking, and electrostatic interactions. A good linear relationship with the malachite green concentration in the range of 1–60 μM was observed with an LOD of 0.30 μM. Similar approach has been published by Li, G. et al. for the determination of malachite green in tap and river water samples [84]. A novel ratiometric sensing strategy was developed using Bio-CDs and Au nanoclusters as dual-emission elements. Blue–green-emitting CDs were synthesized from biomass waste via a one-pot hydrothermal method, and BSA was used as a template to prepare BSA-stabilized gold nanoclusters. While the use of biomass waste and a simple synthesis route is advantageous from a sustainability perspective, the sensing performance demonstrated moderate sensitivity, exhibiting linearity for malachite green over the range of 0.05–40 µM with an LOD of 25 nM, which is comparable to, rather than superior to, many existing fluorescence-based methods.

Zhao, R. et al. proposed a sensing strategy for the determination of Al(III) ions and pH in water samples [91]. The approach is conceptually appealing in enabling dual-parameter analysis. A Zr-based MOF anchored on a rare-earth MOF (UiO-66(OH)2@Y-TCPP) was prepared. A wide linear concentration range of 0.1–1000 μM were recorded with an LOD of 0.06 μM. The fluorescence intensity ratio (I_496_/I_676_) increased markedly, resulting in an observable color change discernible by the naked eye; however, the robustness of this visual response under complex sample conditions was not fully evaluated. Notably, a strong linear relationship was established between the I_496_/I_676_ ratio and Al(III) concentration. The accuracy of the method was investigated at three concentration levels of 20, 100 and 500 μM with RSD values lower than 8.047%. Good recoveries were obtained in the range of 91.0–103%.

Luminescent probes derived from citric acid l-cysteine and cysteamine for instrumental and smartphone-based fluorimetric sensing purposes were studied [79]. Both fluorophores show strong potential for ratiometric fluorimetric pH sensing and are particularly well suited for smartphone-based pH detection through ratiometric analysis using appropriately selected blue and green color channels. The pH-dependent structure–property relationships and the role of the citric acid-derived carboxyl group were elucidated. A Plackett–Burman design was utilized to screen five experimental parameters. Adequate analytical figures of merit were achieved such as a LOD of 37 μM and repeatability (RSD) of 5.8%. Acceptable method accuracy was reported with recoveries ranging from 85% to 106%.

A portable system using polychromatic fluorescent PAD for the quantitative analysis of norfloxacin [92]. The proposed approach was based on a ratiometric sensing platform with an “on–off–on” polychromatic fluorescence response was developed for direct visual and quantitative analysis of norfloxacin. In this system, Fe^3+^-chelated blue CDs functioned as the responsive sensing signal, while red-emissive CDs served as an internal reference. The sensing mechanism was attributed to selective quenching of the blue fluorescence by Fe^3+^ via the inner filter effect. Although a broad linear range of 1–70 μM and a low LOD of 6.84 nM were reported, the reliance on the inner filter effect may limit selectivity under complex sample conditions. Moreover, while satisfactory recoveries (96.53–103.23%) with RSDs below 9.36% were obtained in ultrapure water, tap water, river water, and milk samples, further evaluation of long-term stability and potential interference from coexisting metal ions would be necessary to fully assess practical applicability.

Nitrite is extensively used as a food additive and preservative, in agricultural fertilizers, and as a vasodilator in physiological systems [93]. Wang, C. et al. proposed a smartphone-assisted fluorescence and colorimetric method for nitrite detection based on a paper strip incorporating 2,3-diaminonaphthalene and gold nanoclusters [80]. Under acidic conditions, nitrite reacts with 2,3-diaminonaphthalene via a coupling reaction to form naphthotriazole, which emits strong blue fluorescence at 435 nm, while the red fluorescence of the gold nanoclusters at 650 nm is simultaneously quenched due to nitrite-induced reduction. Although the ratiometric design improves visual discrimination and mitigates signal fluctuations, the method relies on acidic reaction conditions and fluorescence quenching of gold nanoclusters, which may limit robustness and selectivity in complex food matrices. Data acquisition was performed using a Samsung Galaxy S4 CMOS sensor with the Color Grab application under 365 nm UV excitation; however, the dependence on specific smartphone hardware and external UV illumination may constrain broader practical deployment.

Dihydrolipoic acid-stabilized silver nanoclusters with good water solubility were employed as a fluorescent nanoprobe for sulfide ion analysis in tap and drinking water samples [78]. The nanoclusters showed intense red emission at 650 nm upon excitation at 420 nm, which was utilized for fluorometric sulfide sensing. The fluorescence intensity displayed a strong linear correlation with S^2−^ concentrations in the range of 1–15 µM (R^2^ = 0.9948), with a calculated LOD of 32.71 nM. While linearity and sensitivity are acceptable for laboratory-based analysis, the relatively narrow dynamic range may limit applicability for samples with widely varying sulfide levels without additional dilution or calibration steps.

A programmable, printed paper-based device was developed using molybdenum disulfide nanoplates in combination with Eu^3+^ and guanosine 5′-monophosphate and citric acid and applied to the universal detection of TC in various samples (soil, river water, milk, and serum) [94]. The molybdenum disulfide and the guanosine 5′-monophosphate-based nanoparticles were synthesized and combined as composite probe inks. The probes were first characterized morphologically and subsequently validated experimentally for TC sensing. The fluorescence signal of fabricated nanoparticles exhibited high stability across varying pH conditions, while the response of guanosine 5′-monophosphate/Eu^3+^/citric acid remained relatively constant in the pH range of 8–10. When integrated with a custom smartphone application and a 3D-printed measurement chamber, a two-stage programmable printing strategy was implemented to maximize sensing performance. The reproducibility of the method was less than 2.9%. It was found that soil samples contained much higher TC concentrations (0.427–0.815 μM) than other matrices. The obtained results were verified with LC-MS.

Sulfide sensing was performed using paper-assisted ratiometric fluorescent sensors [95]. The sensor was developed using the IFE between CDs and cadmium sulfide QDs. Rather than operating in an aqueous phase, sulfide was converted to its hydride, which induced the in-situ formation of cadmium sulfide QDs directly on the paper substrate. These dots acted as energy acceptors to quench the emission of CDs, resulting in a ratiometric fluorescence change from blue to yellow as the sulfide concentration increased. During fabrication of the portable device, a heating module was incorporated to maintain the temperature at 50 °C and facilitate H_2_S generation. The module was custom designed using a ceramic plate and aluminum alloy to form three grooves compatible with headspace bottles. While this configuration facilitates sample handling, it may limit scalability and adaptability to alternative sample formats. The precision of the developed sensor was assessed using two certified reference materials, and analysis of real water samples yielded recoveries in the range of 88–112%. Although these results indicate reasonable accuracy, the relatively broad recovery range suggests that further optimization and validation across a wider range of matrices would be beneficial.

Recently, a portable 3D-printed platform integrating MIP-coated paper with smartphone-based fluorescence detection was developed for TC sensing [85]. The system employed monochromatic LED strips as an excitation source and a smartphone camera as the detector, with digital image analysis performed using the RGB color model in ImageJ software. Under optimized conditions, an LOD of 40 ppb and good linearity up to 5 ppm (r = 0.998) were reported, with intra- and inter-day precisions of 4.9% and 7.2%, respectively. While the platform demonstrates acceptable analytical performance and portability, its reliance on 3D printing, external excitation components, and post-acquisition image processing may increase system complexity and limit throughput. In addition, although the BAGI tool indicated favorable method applicability, a more comprehensive assessment of material consumption and device reusability would strengthen claims of sustainability. Taken together, these examples highlight the suitability of PADs for low-cost, on-site environmental analysis, while further improvements in detection limits, robustness, and multiplexing capability remain necessary for field deployment.

### 3.4. Pharmaceutical Analysis

Rapid and reliable analysis of fluorescence changes induced by carbazochrome on a PAD was achieved using a smartphone-integrated all-in-one platform featuring a low-cost 365 nm UV excitation source and free image processing software [96]. The principle of the method was based on the reaction of carbazochrome with graphene quantum dots. Image processing was performed using two different software (Color Grab, ImageJ). The excitation and emission wavelengths were 350/440 nm. The LOD was 12 ng/detection zone was reported with acceptable recoveries (100.0 ± 0.4). The sampling rate was sufficient at 16 sample per run. It was proved that the method was selective against other drugs. A score of 0.87 in the AGREE assessment was obtained. The device was described as fully portable, user-friendly, and inexpensive, with an estimated total cost of less than $20. While these features are advantageous for point-of-use applications, the cost estimate does not account for potential expenses associated with calibration, smartphone variability, or replacement of consumable components. The method was applied to the analysis of the drug in pharmaceutical formulations; however, its performance in more complex or less controlled matrices remains to be demonstrated.

Novel strategies were developed for the simultaneous determination of acetaminophen and its potentially toxic impurity, 4-nitrophenol, using both a custom-built in-house device and a conventional benchtop fluorimetric technique [97]. The methods relied on dual excitation of fluorescent graphene QDs at 240 and 350 nm with monitoring of the emission at 440 nm. Although the dual-excitation approach enables concurrent analysis of both compounds, the requirement for deep UV excitation and reliance on a single emission wavelength may restrict selectivity and limit practical implementation, particularly for portable or low-cost platforms. The drug effectively quenched the fluorescence of graphene quantum dots under excitation at 240 nm, whereas 4-nitrophenol induces fluorescence quenching when the GQDs are excited at 350 nm. The quenching mechanism was identified as the inner filter effect, arising from absorption of the incident excitation light by the quenching species. Using the benchtop fluorometric method, acetaminophen and 4-nitrophenol were quantified over concentration ranges of 0.1–20 and 0.1–2.0 µg mL^−1^, respectively. Although both approaches were validated for the analysis of pharmaceutical formulations, the relatively narrow linear range for 4-nitrophenol and the restriction to simple matrices suggest that further evaluation in more complex samples would be necessary to fully demonstrate robustness and broader applicability.

The total flavonol glycosides in *Ginkgo biloba* leaf dropping pills was determined by using a portable ratiometric PAD [98]. The Al^3+^/Eu-MOF paper probe was constructed by immobilizing lanthanide MOF nanoparticles onto Whatman filter paper, followed by coordination with Al^3+^ ions to generate active sensing sites. Detection is based on the hydrolysis of flavonol glycosides to free flavonols, which subsequently coordinate with Al^3+^ through the 3-hydroxyl and 4-keto oxygen groups, forming fluorescent chelate complexes. Flavonoids lacking these specific binding motifs do not participate in complex formation and therefore do not contribute to the fluorescence response. The fluorescence color of the paper substrate gradually changes from red to orange as a function of the flavonol concentration in the sample solution. The selectivity of the method was evaluated against various compounds and metals. The linearity of the method was satisfactory, being in the range of 7–80 μg/mL with an LOD of 2.07 μg/mL. Good method performance was indicated, with recoveries ranging from 93.5 to 113.3% and RSD < 7.2%.

CD nanoprobes were synthesized for the determination of naftazone in its pharmaceutical formulations [99]. The sensing mechanism is governed by an inner filter effect, in which naftazone strongly absorbs in the spectral region overlapping with the excitation (350 nm) and emission (440 nm) bands of the graphene QDs. This spectral overlap attenuates the excitation light reaching the QDs and partially reabsorbs the emitted fluorescence, leading to a concentration-dependent decrease in fluorescence intensity without direct electronic interaction between the analyte and the nanomaterial.

### 3.5. Others

Malhotra, K. et al. proposed a paper-based nucleic acid hybridization assay coupled with smartphone imaging for the detection of cystic fibrosis transmembrane conductance regulator sequences [100]. Assays that function efficiently in aqueous solutions require further optimization for implementation on paper-based substrates due to challenges arising from surface interactions within matrices that possess high surface-to-volume ratios and limited convective mixing. This study presents and compares two related methods for the determination of oligonucleotides used as biomarkers of cystic fibrosis, enabling discrimination between the normal wild-type sequence and a mutant sequence containing a three-base substitution. The signal transduction strategy is based on selective hybridization of oligonucleotide probes conjugated to fluorescent QDs; upon hybridization with the target sequence, a molecular fluorophore is brought into proximity to the QD, resulting in fluorescence resonance energy transfer and signal generation. The LODs achieved were in the range of 215–1450 pmol.

## 4. Conclusions

Fluorescence-based PADs integrated with smartphone imaging constitute a critically important research domain for advancing point-of-need chemical and biological sensing, particularly in resource-limited and field-based settings. Their unique combination of low cost, portability, simplicity, and high analytical performance addresses a growing global demand for rapid, decentralized, and accessible diagnostic and monitoring tools. Recent progress in fluorophore engineering, paper substrate modification, and device architecture has already demonstrated the potential to overcome traditional limitations in sensitivity and selectivity. Ongoing research is now playing a pivotal role in tackling the major remaining challenges of this field, including signal reproducibility, environmental interference, and the lack of standardized analytical protocols. The integration of machine learning-assisted image analysis is expected to significantly enhance signal robustness, accuracy, and automation, while multiplex detection strategies will expand analytical capability for complex real-world samples. Furthermore, coupling PADs with Internet of Things platforms can enable real-time data transmission, large-scale monitoring, and improved decision-making. Together, these advancements highlight the critical significance of this research area and demonstrate how current and future innovations are systematically addressing existing barriers, positioning fluorescence-based PADs as transformative tools for next-generation, reliable, and scalable point-of-need analytical sensing.

## Figures and Tables

**Table 2:** Environmental applications of PAD-based methods.

Analyte	Sample	Method Principle	λ_ex_ (nm)	LOD	Color Processing Application	Ref.
Peracetic acid	Airborne	Oxidative hydroxylation of a phenylboronic acid containing dye.	365	3.5 ppb	ColorMeter RGB Hex	[69]
MnO_4_^−^	Water	Reaction of permanganate with synthesized fluorescent CQDs	400	3.31 μM	–	[70]
Fe(III), Cu(II)	Water	Complexation of Fe(III) and Cu(II) with disalicylaldehyde-based probe	365	0.21, 0.40 μM	–	[71]
2,4,6-trinitrophenol	Tap and river water	Fluorescent quenching of polythiophene-coated CdTe quantum dots	365	0.56 nM	PAD analysis	[72]
Bromine	Water	Luminescence quenching of the probe (5-oxo-3,5-dihydro-2 H-thiazolo[3,2-a]pyridine-7-carboxylic acid)	254/365	5.4 μg/L	App RGB Color Detector	[73]
Copper	Water	The detection mechanism was based on Cu(II)-catalyzed azide-alkyne cycloaddition (Cu(II)AAC) reaction	365	0.1 μM	Pixolor App	[74]
Hg(II)	Water	Reaction of Hg(II) with CQ/Ru(III) nanocomposites	400	95 nM	F Color Picker	[75]
Dipicolinic acid, Cu^2+^	Drinking water	Reaction with bifunctional fluorescent nanoprobe (ATP@Eu:Tb-MOF)	254	6.94 nM	–	[76]
Hg(II)	Water	Reaction of Hg(II) with carbon dots	370	0.23 μM	Color Name	[77]
Malachite green, leuco-malachite green	Fish pond water	Reaction with dual-emissive Eu^3+^-metal–organic framework	302	1.98 nM, 34.2 nM	–	[78]
Ammonia, pH	Water, sendiment	Reaction with citric acid-based cysteine and cysteamine luminescent probes	364	37 μM	–	[79]
Nitrite	Water	Reaction with 2,3-diaminonaphthalene and gold nanoclusters	370	2.3 ppb	Color Grab	[80]
Fe^3+^, ascorbic acid	Water	Reaction with nitrogen-doped CQDs	532	253 nM, 1570 nM	–	[81]
Sulfide	Tap water, drinking water	Reaction of sulfide with dihydrolipoic acid stabilized silver nanoclusters	420	32.71 nM	Color Recognizer	[82]
Cu^2+^	Tap water	Reaction with blue fluorescence CQDs and yellow fluorescence CQDs	450	730 nM	–	[83]
Malachite green	Tap and river water	Reaction with bovine serum albumin-Au dots	350	25 nM	–	[84]
TC		MIP-coated paper	400	40 ppb	ImageJ	[85]

## Data Availability

No new data were created or analyzed in this study. Data sharing is not applicable to this article.

## Abbreviations

The following abbreviations are used in this manuscript:

CD	Carbon dot
CQDs	Carbon quantum dot
FRET	Fluorescence resonance energy transfer
FTIR	Fourier transform infrared
IFE	Inner filter effect
LOD	Limit of detection
LOQ	Limit of quantitation
MIP	Molecularly imprinted polymer
MOF	Metal–organic framework
NCPs	Nanoscale coordination polymers
NIR	Near-infrared
PAD	Paper-based analytical device
PADs	Paper-based analytical devices
POC	Point-of-care
QD	Quantum dot
RSD	Relative standard deviation
SEM	Scanning electron microscopy
TC	Tetracycline
TEM	Transmission electron microscopy
TPCA	5-oxo-3,5-dihydro-2 H-thiazolo[3,2-a]pyridine-7-carboxylic acid
TMB	3,3′,5,5′-tetramethylbenzidine
